# Unexpectedly Low Rate of Metastasis and Death Among Patients Treated for Uveal Melanoma with Brachytherapy, Vitrectomy, and Silicone Oil

**DOI:** 10.3390/cancers17162683

**Published:** 2025-08-18

**Authors:** Axel Rivas, Wolfram Samlowski, Tara A. McCannel

**Affiliations:** 1 Kirk Kerkorian School of Medicine, University of Nevada, Las Vegas (UNLV), Las Vegas, NV 89102, USA; rivasa9@unlv.nevada.edu; 2 Nevada Oncology Specialists, Las Vegas, NV 89148, USA; 3 Jules Stein Eye Institute, Department of Ophthalmology, University of California, Los Angeles, Los Angeles, CA 90095, USA

**Keywords:** ocular melanoma, uveal melanoma, gene expression profiling, monosomy 3, cytogenetics, uveal melanoma metastasis, uveal melanoma survival, brachytherapy, vitrectomy, silicone oil

## Abstract

**Introduction:** Historically, approximately 30–32% of uveal melanoma patients develop metastases and die within 5 years. Larger tumor dimensions and monosomy 3 and/or Class 2 gene expression profile of the tumor are associated with an increased risk of metastasis. We evaluated the performance of these prognostic markers in patients undergoing surveillance in a community medical oncology practice. **Methods:** Uveal melanoma patients treated by a single vitreoretinal surgical oncologist and monitored by a single medical oncologist were identified via a clinical database search for analysis. Specified prognostic markers were evaluated for sensitivity, specificity, positive and negative predictive value, as well as prognostic accuracy. **Results:** We identified 37 uveal melanoma patients, followed for a median of 4.0 ± 3.7 years. Twenty-seven (73%) patients underwent plaque brachytherapy with vitrectomy and silicone oil for radiation attenuation; seven (19%) patients underwent brachytherapy alone; three (8%) had enucleation. There were no intraocular treatment failures. Six (16.2%) patients developed metastasis, and only one patient died of metastatic disease. Disomy 3 and Class 1 accurately predicted a low risk for metastasis (>90% negative predictive value). However, monosomy 3 and Class 2 had a low positive predictive value and prognostic accuracy in “high-risk” patients. **Conclusions:** In this single-center cohort, we observed an unexpectedly low rate of 5-year metastasis. AJCC stage correlated poorly with metastasis or survival. Molecular prognostic results of Disomy 3 and Class 1 gene expression profile accurately predicted a “low” risk for metastasis (>90% negative predictive value). Further evaluation of these unique surgical techniques needs to be carried out.

## Simple Summary

Patients with melanoma arising in the eye have a high risk of spreading to the liver and other organs. When this occurs, cancer-related death is frequent. We evaluated 37 recently treated eye melanoma patients in our melanoma-specialized practice. All patients had been treated by a single ocular oncologist. Most patients were treated with plaque radiation and replacement of the gel-like content of the eye (vitreous) with silicone oil to reduce radiation effects on vision. Patients were monitored for over 4 years, on average. The rate of cancer spread was lower than expected. No cancers recurred in the eye. Only six patients developed cancer that spread to the liver, and only one of these patients died. Testing designed to identify patients at “low risk” for metastasis was accurate. However, most patients who were classified as “high risk” remained free of metastasis. Development of more accurate testing methods is needed.

## 1. Introduction

Uveal melanoma is a rare malignancy that represents 85% of malignant intraocular tumors in adults [1]. The overall incidence is approximately 5.1 cases per million persons per year in the United States [2]. There are significant racial influences that govern the development of uveal melanoma. Nordic populations have a higher incidence of uveal melanoma than Asians and Africans [3]. While there appears to be a relationship between the incidence of uveal melanoma, ocular pigmentation, and sun exposure, the pattern of DNA damage in genomic studies suggests an indirect mechanism of carcinogenesis, rather than direct UV-induced damage to retinal DNA [4].

The current standard management of localized uveal melanoma is globe-conserving treatment with brachytherapy rather than enucleation. The Collaborative Ocular Melanoma Study (COMS) demonstrated equivalent survival following both treatment approaches [5]. Historically, 52% of uveal melanomas developed metastases within 35 years from treatment of the ocular primary [6]. In this era, the overall 10-year mortality of uveal melanoma was approximately 43% [6,7]. 

Unlike most cancers, uveal melanoma has an increased predilection for the development of hepatic metastases as the main site of distant progression [8]. Another unusual feature of uveal melanoma is the potential for delayed metastatic occurrence many years or even decades following treatment of the ocular primary tumor [9,10]. Historically, the prognosis was poor once metastases developed, with a median survival of only 10–13 months [7].

There have been recent attempts to stratify patients into “high-risk” and “low-risk” populations, using potential prognostic biomarkers, such as clinical tumor (T) stage, tumor cytogenetics (e.g., monosomy 3), and immunohistochemistry (BAP-1 mutation testing) [11,12], and gene expression profile (GEP) testing [13].

The goal of the present study was to evaluate the current clinical outcomes of uveal melanoma treatment in a community practice. An exploratory analysis of clinical stage, monosomy 3, and GEP testing in predicting the risk of distant metastasis-free survival (DMFS) and overall survival was performed.

## 2. Materials and Methods

### 2.1. Subjects and Design

Potential records for review were identified by searching for patients under the care of a single oncologist (WS) with a diagnosis of uveal (choroidal) melanoma. Only patients whose ocular tumors were treated by a single vitreoretinal surgical oncologist (TAM) were included. Data was stored in a Health Information Portability and Accessibility Act (HIPAA) compliant clinical oncology database (IKnowMed, McKesson, Houston, TX, USA). Individual patient records were accessed to extract clinical data into a password-protected spreadsheet. Data extracted for analysis included demographics and tumor characteristics. Baseline uveal melanoma tumor size (largest basal diameter and height) was used to define AJCC (8th Edition) tumor (T) stage [14]. Monosomy 3 cytogenetics and GEP testing (Castle Bioscience DecisionDx Uveal Melanoma test, Friendswood, TX, USA) were performed on a fine needle aspiration tumor biopsy collected at the time of treatment. A unique patient number was assigned to each patient. Subsequently, patient data was deidentified prior to analysis. This study design was reviewed by the Western Institutional Review Board (IRB) chair and deemed exempt from full IRB review.

### 2.2. Risk Stratification

To analyze the differences in DMFS and overall survival, smaller tumors (combined T1 and T2 stages) were compared to larger tumors (combined T3 and T4 stages). Based on cytogenetic testing of the intraocular tumor, patients were evaluated for the presence of monosomy or Disomy 3 and/or the tumor GEP score. To be characterized as “high-risk”, patients expressed either monosomy 3 or a Class 2 GEP score, or both. In contrast, “low-risk” patients had Disomy 3 or a Class 1A or 1B GEP score. To emphasize the potential inaccuracy of the ability of current prognostic testing to identify patients who will eventually metastasize, we have placed “high-risk” and “low-risk” in quotation marks.

### 2.3. Treatment of Ocular Primary

Patients diagnosed with uveal melanoma underwent brachytherapy plaque alone, brachytherapy plaque with vitrectomy and silicone oil for radiation attenuation, or enucleation. Technical details of brachytherapy and vitrectomy techniques have been published elsewhere [15,16,17].

### 2.4. Post-Treatment Surveillance

Following primary uveal melanoma treatment, patients were monitored for the development of liver metastases on the following schedules: “Low-risk” (Disomy 3 or Class 1 GEP) patients were monitored with lactate dehydrogenase (LDH) and aspartate and alanine aminotransferases (AST/ALT), clinical examination and liver imaging [ultrasound or liver magnetic resonance imaging (MRI)] every 6 months for 2 years. “High-risk” (monosomy 3 or Class 2 GEP) patients underwent evaluation with similar testing every 3 months for two years. Subsequently, “low-risk’’ patients were monitored annually, and “high-risk” risk patients were monitored every 6 months. After 5 years, all patients were monitored annually with blood tests as above and abdominal imaging.

### 2.5. Outcome Assessment

Metastasis-free survival as well as overall survival were calculated from the date of initial treatment (brachytherapy or enucleation) to the date of metastasis or death. If patients were metastasis-free or alive at the last date of follow-up, the patient was censored at the date of the last clinic visit. Overall survival and metastasis-free survival were analyzed using methods described by Kaplan and Meier [18]. The end date of the current analysis was 12 July 2023. Statistical comparison of patient subsets was performed using the log-rank test (GraphPad Prism software, Software McKiev, Boston, MA, USA, Version 10.2.0). 

### 2.6. Diagnostic Test Performance Analysis

The data on tumor size, monosomy 3, or Class 1/2 GEP score were utilized to evaluate prognostic test performance. The main objective was to estimate the sensitivity, specificity, positive and negative prognostic value, and accuracy of each assay to predict uveal melanoma metastasis and mortality [19,20]. This was achieved through statistical software (MedCalc Software Ltd., Ostend, Belgium, Version 22).

### 2.7. Sensitivity

Sensitivity was defined as the proportion of patients with monosomy 3, Class 2 GEP, or initial T3–4 clinical staging who eventually progressed to metastasis or death. True positives (TP) were patients with a positive test result who progressed or died, while false negatives (FN) were individuals who were stratified as low risk and progressed to metastasis or death. Sensitivity was calculated from the formula TP/(TP + FN) (MedCalc Software Ltd., Ostend, Belgium, Version 22). 

### 2.8. Specificity

Specificity was utilized to assess the accuracy of markers suggesting a low recurrence risk, such as Disomy 3 or a Class 1A or 1B GEP. True negatives (TN) were individuals with a “low-risk” score who did not have a cancer recurrence or death. False positives (FP) were individuals with “high-risk” predictors who did not progress to death or metastasis. Specificity was calculated from the formula TN/(TN + FP) (MedCalc Software Ltd., Ostend, Belgium, Version 22).

### 2.9. Positive Prognostic Value (PPV)

The PPV represents the proportion of patients with a “high-risk” test result (monosomy 3, Class 2 GEP, or high initial T stage) who eventually developed metastasis. False positives were patients who had a “high-risk” test result without tumor progression. Positive prognostic value was calculated from the formula TP/(TP + FP) (MedCalc Software Ltd., Ostend, Belgium, Version 22). 

### 2.10. Negative Prognostic Value (NPV)

The NPV indicates the percentage of patients with a “low-risk” test result (Disomy 3, Class 1A or 1B GEP, or low initial T stage) who did not eventually develop tumor progression. These patients were considered to have a true negative test result (TN). Patients with a “low-risk” score who eventually developed metastasis were considered false negatives (FN). The negative prognostic value was calculated from the formula TN/(TN + FN) (MedCalc Software Ltd., Ostend, Belgium, Version 22). 

### 2.11. Prognostic Accuracy

Prognostic accuracy was calculated from the formula (TP + TN)/(TP + TN + FP + FN). A high prognostic risk accuracy value would mean that a test is effective at distinguishing between patients who are at “high-risk” of progression and correctly identifies “low-risk” patients. Accuracy is compromised by a high number of false positives or false negatives (MedCalc Software Ltd., Ostend, Belgium, Version 22).

### 2.12. Confidence Intervals

Confidence intervals for sensitivity, specificity, and accuracy were calculated using the exact Clopper–Pearson method through MedCalc statistical software (MedCalc Software Ltd., Ostend, Belgium, Version 22). Confidence intervals for negative and positive prognostic values were obtained using standard logistic regression via the MedCalc software.

## 3. Results

### 3.1. Demographics

A total of 37 patients with uveal melanoma treated by a single vitreoretinal surgical oncologist (TAM) were identified from a computer search of medical records. These patients were systematically monitored by a single medical oncologist (WS) in a community practice. Individual patient characteristics are provided (Supplemental Table S1). Patient ages ranged from 24 to 84 years, with a median age of 59 ± 12.5 years. Most patients were male (70%) and non-Hispanic Caucasians (90%)**.** Among the 37 patients, treatment modalities included brachytherapy plaque alone in 7 patients (19%), brachytherapy plaque with vitrectomy and silicone oil for radiation attenuation in 27 patients (73%), and enucleation in 3 patients (8%).

### 3.2. Tumor Dimensions

Tumor height and maximum basal diameter were recorded for each patient. Tumor height ranged from 1.14 to 14.92 mm (median 4.06 mm). The maximum basal diameter ranged from 6.88 to 21.62 mm (median 13.0 mm). Individual tumor dimensions for each patient in this cohort are provided (Supplemental Table S1).

### 3.3. Analysis of DMFS and Overall Survival

**Figure 1:**
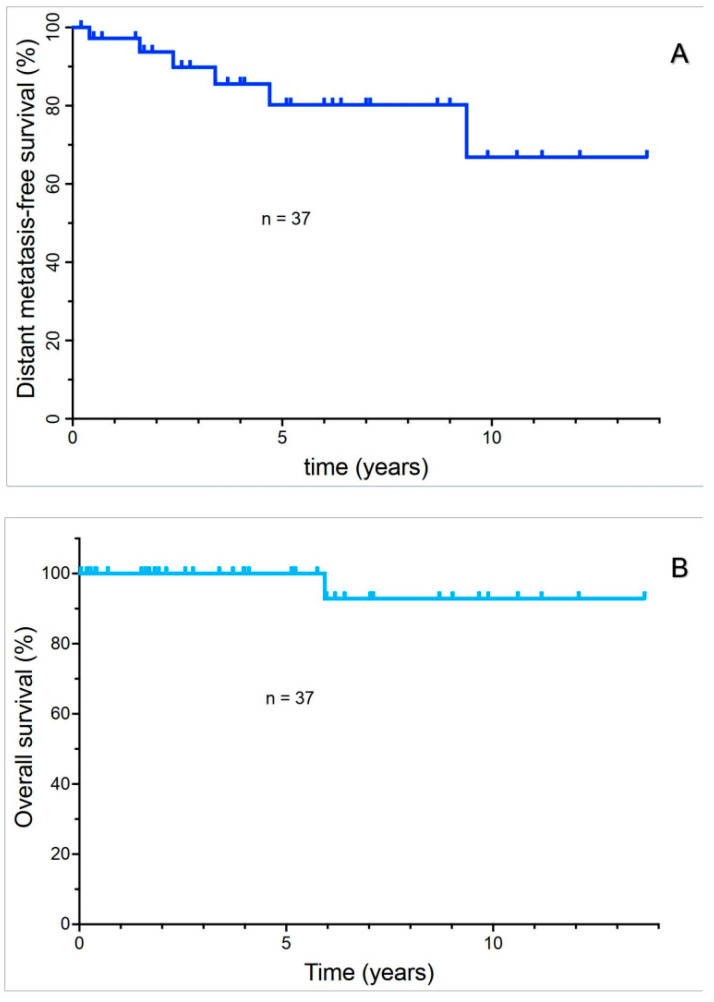
Distant metastasis-free survival of uveal melanoma patients (**A**); Overall survival of uveal melanoma patients (**B**).

With a median follow-up of 4.0 ± 3.7, DMFS at 5 years was 80%. The median DMFS was not reached (Figure 1A). Metastatic disease eventually occurred in six patients (16.2%). There were no patients who developed intraocular treatment failure. Overall survival at 5 years remained at 100% (Figure 1B). Only one patient (2.7%) died from metastatic disease prior to the date of analysis. Thus, overall survival was not further analyzed in this study as an endpoint.

### 3.4. Exploratory Analysis of the Relationship Between DMFS and AJCC Stage

**Figure 2:**
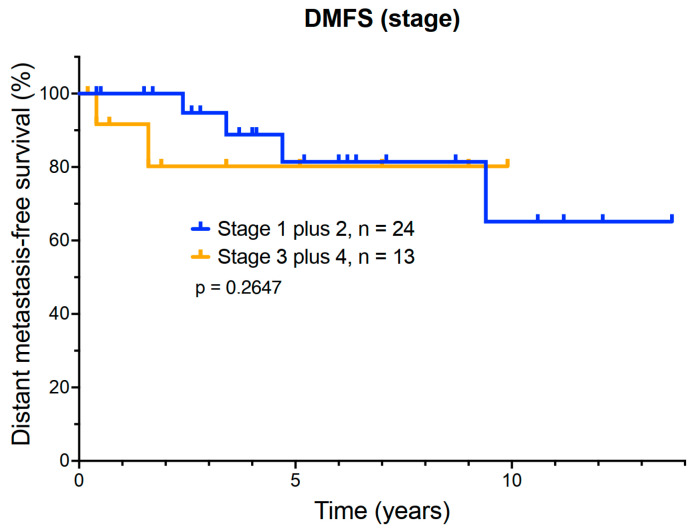
Distant metastasis-free survival of T1 and 2 stage patients versus T3 and 4 uveal melanoma patients.

**Figure 3:**
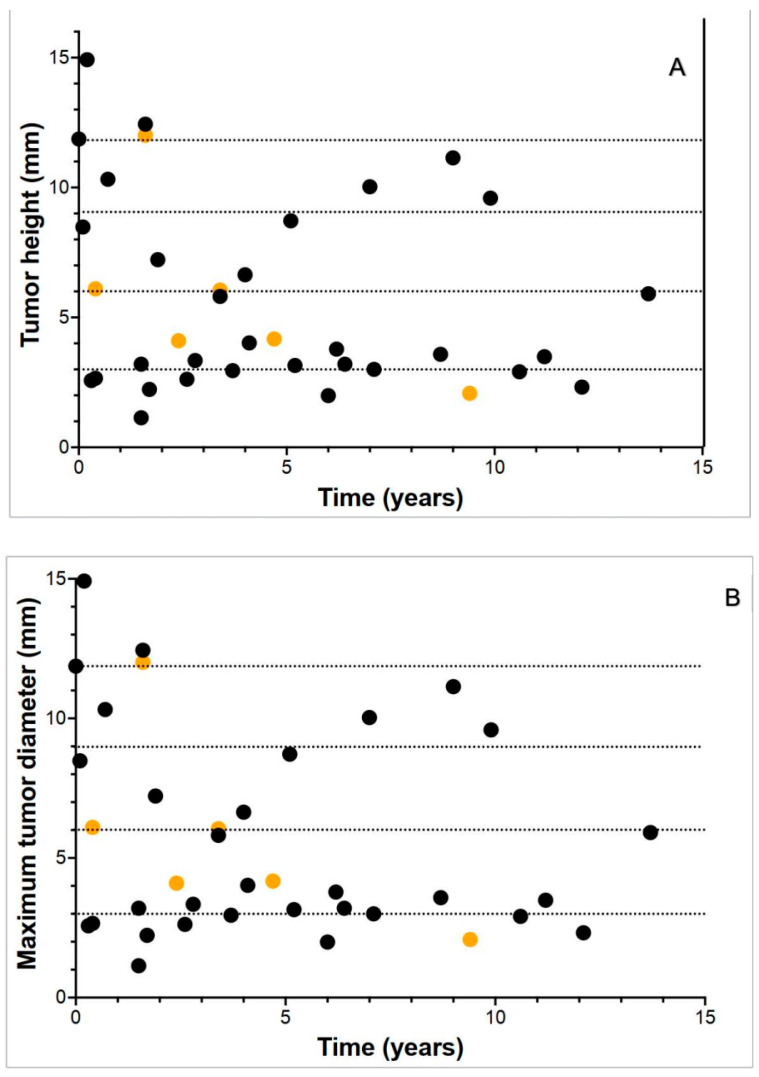
Relationship of intraocular tumor dimensions to distant metastasis-free survival. The relationship to tumor height is shown (**A**); the relationship to the maximum tumor diameter is depicted (**B**). Relapsed patients are indicated in orange. The horizontal dotted lines indicate the size criteria utilized in the current AJCC8 staging system to assign the T stage.

Bidimensional primary tumor measurements were available for 31 of the 37 patients. The tumor stage was as follows: 15 patients had T1 tumors (40.5%); 9 patients had T2 tumors (24.3%); 8 patients had T3 tumors (21.6%); and 5 patients had T4 tumors (13.5%). Due to small patient numbers, patients with T1 and T2 tumors were grouped together for analysis, as were patients with T3 and T4 tumors. There were 28 patients in the T1 and T2 group, while 17 patients had either T3 or T4 lesions. DMFS by clinical stage is shown (Figure 2). Comparison of DMFS between T1–2 and T3–4 stage patients did not identify a significant difference in outcome. Even patients with large intraocular primary tumors (T3–4) had an estimated 5-year survival of 85%. The relationship between tumor height and maximal basal diameter and DMSF was also plotted individually for each patient (Figure 3A,B). This analysis further emphasized the dissociation between tumor measurements and metastatic risk. 

Of the six patients that eventually developed metastasis, five had stage T1 or T2 tumors, while only one patient with a T4 tumor progressed. This counterintuitive result reflects the limited predictive value of tumor size alone in AJCC T staging, without consideration of molecular prognostic markers.

### 3.5. Exploratory Analysis of Molecular Prognostic Testing

**Figure 4:**
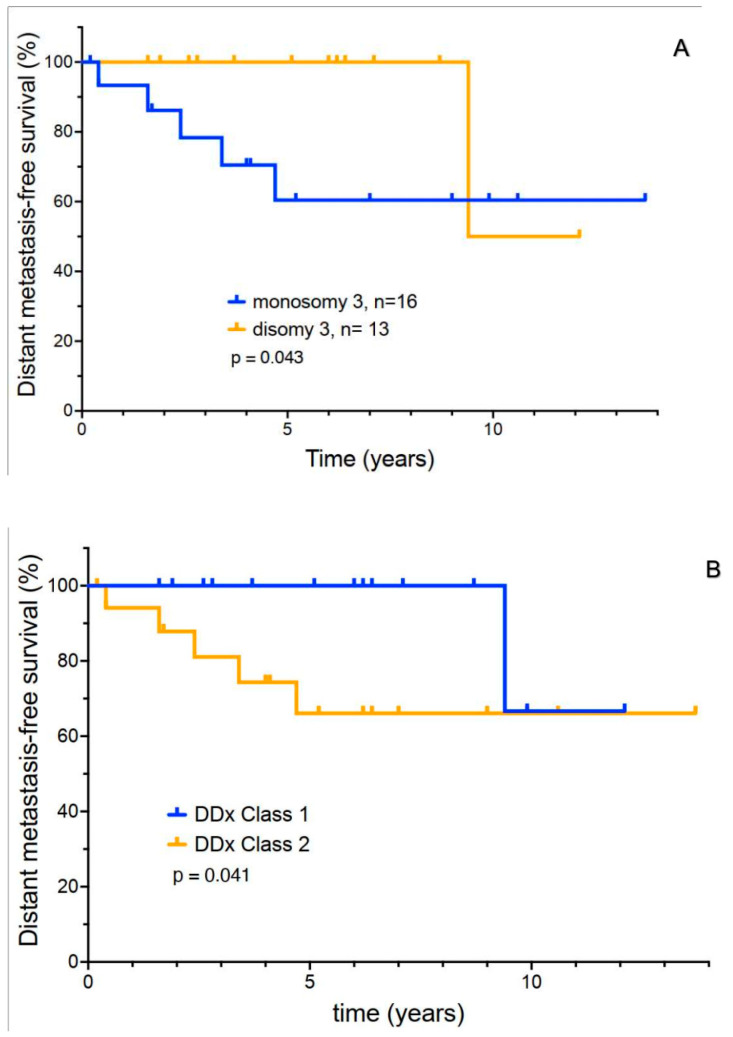
Distant metastasis-free survival of disomy 3 and monosomy 3 uveal melanoma patients (**A**). Progression-free survival for DecisionDx UM class I versus class II uveal melanoma patients (**B**).

It should be noted that prognostic testing methods have evolved over the period of our study. Twenty-nine patients had cytogenetic testing of the intraocular primary for monosomy 3. Sixteen had monosomy 3, and thirteen had disomy 3. A Kaplan–Meier plot of DMFS for both groups is shown (Figure 4A). A total of 5 of 16 patients with monosomy 3 developed metastasis. Only one patient with disomy 3 progressed to metastatic disease (*p* = 0.043). It is thought that the tumor suppressor BAP-1 is deleted in monosomy 3. Sixteen patients in our cohort underwent analysis of BAP-1 protein expression using immunohistochemistry. It is counterintuitive that none of the patients with BAP-1 deletions (0/7) developed metastases. Of nine patients with normal BAP-1 expression, three developed metastatic recurrence. Thus, this potential prognostic test was not subjected to further analysis due to poor correlation with outcome.

Sixteen patients underwent Decision DX UM gene expression profiling. Seven patients were assigned to Class 1A, one patient was in Class 1B, and eight patients were in Class 2. One patient with a Class 1A or B GEP developed metastasis over the course of the study. Two of eight Class 2 patients developed eventual metastasis (Figure 4B) (*p* = 0.041). It should be noted, however, that median DMFS in Class 2 patients remained 65% at 5 years.

Of the patients who developed distant metastases, 5/6 patients were found to have monosomy 3, and 2/2 had a Class 2 GEP. There was a trend towards earlier recurrence in these six patients (median ~2 years). However, median OS was quite prolonged (~4 years), despite the development of metastatic disease. Five of these patients were still alive and under treatment at the time of this report.

### 3.6. Analysis of the Accuracy of Prognostic Testing

The performance of prognostic markers for each of the three prognostic parameters is presented (Table 1). Tests evaluated included TNM Stage (*n* = 37), monosomy 3 (*n* = 16), and Class 1 or 2 GEP (*n* = 16). Analysis metrics included sensitivity, specificity, positive and negative prognostic value, and diagnostic accuracy for each test [19,20]. Additional test characterization is provided (Supplemental Tables S2–S5).

## 4. Discussion

Uveal melanoma is a rare intraocular malignancy characterized by a significant risk for hepatic metastasis [8]. The current standard management of localized uveal melanoma is globe-conserving treatment with brachytherapy, and enucleation for large tumors that may not be treatable. The Collaborative Ocular Melanoma Study (COMS) demonstrated equivalent survival following both treatment approaches [5]. As a result, survival of uveal melanoma has remained relatively constant over several decades between 1989 and 2019 [2,21]. 

Historically, the size of the intraocular tumor at presentation (measurement of the largest basal diameter and tumor height) provided an important prognostic determinant [14,22]. A large single-center study correlating long-term outcome based on tumor size suggested that every millimeter of increased tumor thickness resulted in an approximately 5% increase in metastatic risk at 10 years [22,23]. Five-year mortality of tumors with the greatest basal dimension, less than 10 mm, was only 8%. Tumors with the greatest basal dimension between 10 and 15 mm had a 32% mortality, and larger-sized tumors with the greatest basal diameter over 15 mm had a 50% mortality at 5 years [23]. Multivariate analysis of the outcome of 5036 patients from the Netherlands has suggested that the survival of uveal melanoma may be improving following treatment of the ocular primary with brachytherapy [21].

These direct tumor measurements are the basis for the current American Joint Commission on Cancer (AJCC) T (Tumor) stage categories (T1–4) [24]. In the data set supporting the AJCC Classification system, the 10-year rate of metastatic disease was 15%, 25%, 49% and 63% for T1, T2, T3, and T4 tumors, respectively. [24].

Our current retrospective analysis was designed to evaluate the clinical outcome of more recently treated uveal melanoma patients monitored in a community oncology practice. All patients were consistently treated for their ocular primary by a single vitreoretinal oncologist. Patients were then systematically monitored for metastatic progression. With a median follow-up of over 5 years, 31/37 of our patients (83.8%) remained metastasis-free. Only six patients (16.2 %) eventually developed evidence of metastatic disease, and only one of these six patients died during the follow-up period. 

Given the surprisingly lower than usual rate of metastasis in this specific community population, it is imperative to consider possible reasons for the superior improvement in metastatic outcome of our patient cohort. 

The unique features of our study population were the following: the majority (73%) of patients underwent treatment with plaque brachytherapy with silicone oil for radiation attenuation; each patient was a resident of the state of Nevada; each patient was under the care of the same medical oncologist (WS); each patient was under the care of the same ocular oncologist (TAM). 

The most common globe-sparing treatment for uveal melanoma is a brachytherapy plaque. Newer vision-saving surgical techniques using vitreoretinal surgery to shield non-tumor parts of the retina with the denser silicone oil 1000 centistokes have been reported and performed for over a decade by the vitreoretinal surgical oncologist in this report. Although we have reported vision-improving benefits using silicone oil for radiation shielding, the impact of these surgical techniques and the impact of improving vision on metastasis may be an entirely novel benefit. Perhaps removing the vitreous humor in uveal melanoma eyes reduces intraocular tumor burden, which may reduce hematogenous spread. Perhaps the use of silicone oil 1000 centistokes has a tumoricidal effect in addition to the radiation therapy from brachytherapy that improves metastatic outcomes. Further laboratory studies may be needed to elucidate these potential additional benefits, which go beyond vision improvement alone.

Unfortunately, a markedly delayed onset of metastases many years or even decades after treatment of the ocular primary is not uncommon [9,10]. This implies that tumor cell dissemination prior to effective local control of the intraocular primary via brachytherapy or enucleation remains a significant clinical challenge. Further work is needed to better identify factors responsible for delayed-onset or the lack of development of metastasis. An improved understanding of the mechanisms underlying the potential prolonged dormancy and eventual reactivation of uveal melanoma cells in the liver is needed. This information may lead to the development of better treatment approaches to eradicate this reactivation of quiescent tumor cells.

We performed an exploratory analysis of potential prognostic markers in our patients, including intraocular tumor stage, as well as cytogenetic monosomy 3 and GEP testing to determine whether these tests could identify patients at risk for early recurrence. In our patient series, AJCC staging correlated poorly with metastatic outcome. Of our patients with small (T1 and T2) intraocular tumors, 5/28 (17.9%) eventually progressed to metastatic disease, compared to only 2/17 (11.8%) with larger (T3 and T4) tumors. Thus, the tumor T stage correlated poorly with the outcome. 

More recently, molecular prognostic tests have been developed to try to identify patients at elevated risk for metastasis. These tests are usually performed on cytologic samples of tumors obtained at the time of brachytherapy surgery. Deletion of one copy of chromosome 3 (monosomy 3) in uveal tumor cells has been associated with an increased risk for metastasis in uveal melanoma [12,22]. This may be related to the loss of the tumor suppressor gene, BAP-1 [25], which may increase melanoma invasiveness and potential for metastasis [12,22,26,27]. A previous study suggested that the 3-year survival rate with monosomy 3 was only 58%, compared to a 100% survival in patients with Disomy 3 [12]. In our current patient series, patients with monosomy 3 had an increased recurrence risk. However, despite the detection of monosomy 3, the 5-year DMFS of patients remained surprisingly high (60.4%) at 5 years. 

The genomic landscape of uveal melanoma appears to be characterized by a small number of recurrent somatic mutations. These observations have resulted in the development of molecular tests such as gene expression profiling (GEP) as potential tools to predict metastatic risk. For example, the Decision Dx UM tests segregate uveal melanoma patients into two groups: Class 1, with a low metastatic risk, and Class 2, with an elevated metastatic risk [28,29]. Retrospective studies evaluating the Decision Dx UM genetic expression panel showed that Class 2 patients had a 3-year DMFS rate of 63% compared to 100% in Class I patients [30,31]. Our patient series, with longer follow-up, confirmed the separation of distant metastasis-free survival curves between Class 1 and 2 patients. Patients with a Class 1 score had a lower recurrence risk. However, the 5-year progression-free survival of our “high-risk” (Class 2) patients was 66.1%. In addition, the time to the onset of metastases in even “high-risk” patients was often lengthy.

In contrast, our exploratory analysis, both cytogenetic and GEP testing, had only a modest positive predictive value. This may be because the risk of distant metastases in our patient population appeared low compared to historical series. Another important point is that even in “high-risk” patients, early recurrence in the first year or two after diagnosis and treatment was rare in our patient series. The metastatic risk was spread out over many years. The fact that most patients with monosomy 3 or a Class 2 GEP score never experienced metastatic disease indicates that these markers do not specifically identify mechanisms that trigger uveal melanoma progression. Further preclinical and clinical investigation is needed to better understand these processes.

The strengths of this study are that we report on a relatively long follow-up interval of 4 years of a relatively large cohort of patients who received systemic surveillance in a community practice setting. All were treated in a consistent fashion by a single vitreoretinal oncologist and monitored in a consistent fashion by the same medical oncologist. Consistent follow-up of this cohort allowed for the collection of both long-term ocular and metastatic outcomes. Our study is original in that it offers a real-world perspective by gathering clinical data from a community setting rather than a quaternary medical center. Therefore, the findings may be more relevant than studies performed at academic institutions. Additionally, our study includes mostly patients who had unique vision-saving vitreoretinal surgical interventions involving silicone oil for radiation shielding, for which metastatic data is not yet known.

Our study is limited by the retrospective nature of the data collection. Additionally, uveal melanoma is a rare cancer that precludes a large patient sample size for analysis. Another limitation of our study is that not all patients had both cytogenetic monosomy 3 and GEP testing performed. Since practice patterns evolved over the years of this study, only 16 more recent patients had GEP data. This study also did not include other prognostic markers, such as BAP-1 immunohistochemistry, as this was not consistently performed and seemed to correlate poorly with outcome. Treatment approaches also varied somewhat over time, with the more recent addition of vitrectomy and silicone oil replacement. These factors may limit the generalizability to the broader international population of patients with uveal melanoma. As a consequence, our study should be considered hypothesis-generating. Confirmation in prospective randomized trials will be required. 

## 5. Conclusions

Uveal melanoma has historically had a poor survival outcome, with a high rate of metastasis. Even when metastatic disease was detected, systemic treatments were minimally effective. One important conclusion to be drawn from our patient sample is that the outcome of brachytherapy plus vitrectomy-treated patients may influence the onset of metastases. Molecular prognostic testing has the potential to identify patients at lower risk for metastasis. This may allow de-escalation of the frequency of screening in “low-risk” patient populations. This possibility requires further prospective evaluation. However, the development of more effective systemic therapies to prevent hepatic seeding by uveal tumor cells or to effectively treat metastatic disease once it develops remains of paramount importance. Although we observed a low metastatic rate in patients treated with plaque brachytherapy, vitrectomy, and silicone oil placement, it is not clear how these procedures may have resulted in improved outcomes. Further laboratory and more rigorous clinical investigations are needed to evaluate whether vitrectomy and silicone oil exert tumoricidal or immunomodulatory effects in a manner that impacts systemic progression or whether more effective brachytherapy delivery alone is responsible for the observed outcome.

## Figures and Tables

**Table 1:** Predictive test summary.

	T Stage (*n* = 37)	Monosomy 3 (*n* = 29)	GEP (*n* = 16)
**Sensitivity**	33.33% (CI 4.3–77.7%)	83.33% (CI 35.9–99.6)	100% (CI 15.8–100)
**Specificity**	64.52% (CI 45.4–80.8)	52.17% (CI 30.6–73.2)	57.14% (CI 28.9–82.3)
**Positive Predictive Value**	15.38% (CI 5.1–38.3)	31.25% (CI 20.7–44.2)	25.0% (CI 15.4–37.9)
**Negative Predictive Value**	83.33% (CI 72.8–90.3)	92.31% (CI 65.8–98.7)	100% (CI 63.1–100)
**Diagnostic Accuracy**	59.46% (CI 42.1–75.3)	58.62% (CI 38.9–76.5)	62.5% (CI 35.4–84.8)

Clinical staging based on tumor dimensions (AJCC T stage) proved to have a very poor sensitivity and a low prognostic accuracy. Both monosomy 3 testing and GEP testing had a high negative predictive value (i.e., these tests accurately predicted patients at low risk for relapse). Unfortunately, the identification of “high-risk” patients suffered from a low positive predictive value and, therefore, prognostic accuracy.

## Data Availability

De-identified primary data will be made available upon reasonable request to the corresponding author.

## Supplementary Materials

The following supporting information can be downloaded at: https://www.mdpi.com/article/10.3390/cancers17162683/s1. Table S1: Individual patient characteristics; Table S2: Demographic Summary: Table S3: Characteristics by T Stage; Table S4: Characteristics by chromosome 3 cytogenetics; Table S5: Patient characteristics by Decision DX UM score.

## References

[1] Chang A.E., Karnell L.H., Menck H.R. (1998). The National Cancer Data Base report on cutaneous and noncutaneous melanoma: A summary of 84,836 cases from the past decade. The American College of Surgeons Commission on Cancer and the American Cancer Society. Cancer.

[2] Singh A.D., Turell M.E., Topham A.K. (2011). Uveal melanoma: Trends in incidence, treatment, and survival. Ophthalmology.

[3] Ortega M.A., Fraile-Martinez O., Garcia-Honduvilla N., Coca S., Alvarez-Mon M., Bujan J., Teus M.A. (2020). Update on uveal melanoma: Translational research from biology to clinical practice (Review). Int. J. Oncol..

[4] Chalada M., Ramlogan-Steel C.A., Dhungel B.P., Layton C.J., Steel J.C. (2021). The Impact of Ultraviolet Radiation on the Aetiology and Development of Uveal Melanoma. Cancers.

[5] Collaborative Ocular Melanoma Study Group (2006). The COMS randomized trial of iodine 125 brachytherapy for choroidal melanoma: V. Twelve-year mortality rates and prognostic factors: COMS report No. 28. Arch. Ophthalmol..

[6] Rantala E.S., Hernberg M.M., Piperno-Neumann S., Grossniklaus H.E., Kivela T.T. (2022). Metastatic uveal melanoma: The final frontier. Prog. Retin. Eye Res..

[7] Rantala E.S., Hernberg M., Kivela T.T. (2019). Overall survival after treatment for metastatic uveal melanoma: A systematic review and meta-analysis. Melanoma Res..

[8] Lamas N.J., Martel A., Nahon-Esteve S., Goffinet S., Macocco A., Bertolotto C., Lassalle S., Hofman P. (2021). Prognostic Biomarkers in Uveal Melanoma: The Status Quo, Recent Advances and Future Directions. Cancers.

[9] Kujala E., Makitie T., Kivela T. (2003). Very long-term prognosis of patients with malignant uveal melanoma. Invest. Ophthalmol. Vis. Sci..

[10] Kujala E., Damato B., Coupland S.E., Desjardins L., Bechrakis N.E., Grange J.D., Kivela T. (2013). Staging of ciliary body and choroidal melanomas based on anatomic extent. J. Clin. Oncol..

[11] Aoude L.G., Vajdic C.M., Kricker A., Armstrong B., Hayward N.K. (2013). Prevalence of germline BAP1 mutation in a population-based sample of uveal melanoma cases. Pigment. Cell Melanoma Res..

[12] Prescher G., Bornfeld N., Hirche H., Horsthemke B., Jockel K.H., Becher R. (1996). Prognostic implications of monosomy 3 in uveal melanoma. Lancet.

[13] Miguez S., Lee R.Y., Chan A.X., Demkowicz P.C., Jones B., Long C.P., Abramson D.H., Bosenberg M., Sznol M., Kluger H. (2023). Validation of the Prognostic Usefulness of the Gene Expression Profiling Test in Patients with Uveal Melanoma. Ophthalmology.

[14] Kivela T., Simpson E.R., Grossniklaus H.E., Jager M.J., Singh A.D., Caminal J.M., Pavlick A.C., Kujala E., Coupland S.E., Finger P.T., Amin M.B., Edge S., Greene F., Byrd D.R., Brookland R.K., Washington M.K., Gershenwald J.E., Compton C.C., Hess K.R., Sullivan D.C. (2017). Uveal Melanoma. AJCC Cancer Staging Manual.

[15] Oliver S.C., Leu M.Y., DeMarco J.J., Chow P.E., Lee S.P., McCannel T.A. (2010). Attenuation of iodine 125 radiation with vitreous substitutes in the treatment of uveal melanoma. Arch. Ophthalmol..

[16] McCannel T.A., McCannel C.A. (2014). Iodine 125 brachytherapy with vitrectomy and silicone oil in the treatment of uveal melanoma: 1-to-1 matched case-control series. Int. J. Radiat. Oncol. Biol. Phys..

[17] McCannel T.A., Kamrava M., Demanes J., Lamb J., Bartlett J.D., Almanzor R., Chun M., McCannel C.A. (2016). 23-mm iodine-125 plaque for uveal melanoma: Benefit of vitrectomy and silicone oil on visual acuity. Graefes Arch. Clin. Exp. Ophthalmol..

[18] Kaplan E.L., Meier P. (1958). Nonparametric Estimation from Incomplete Observations. J. Am. Stat. Assoc..

[19] Trevethan R. (2017). Sensitivity, Specificity, and Predictive Values: Foundations, Pliabilities, and Pitfalls in Research and Practice. Front. Public Health.

[20] Simundic A.M. (2009). Measures of Diagnostic Accuracy: Basic Definitions. Electron. J. Int. Fed. Clin. Chem. Lab. Med..

[21] Tong T.M.L., Bastiaannet E., Speetjens F.M., Blank C.U., Luyten G.P.M., Jager M.J., Marinkovic M., Vu T.H.K., Rasch C.R.N., Creutzberg C.L. (2023). Time Trends in the Treatment and Survival of 5036 Uveal Melanoma Patients in The Netherlands over a 30-Year Period. Cancers.

[22] Kaliki S., Shields C.L. (2017). Uveal melanoma: Relatively rare but deadly cancer. Eye.

[23] Shields C.L., Furuta M., Thangappan A., Nagori S., Mashayekhi A., Lally D.R., Kelly C.C., Rudich D.S., Nagori A.V., Wakade O.A. (2009). Metastasis of uveal melanoma millimeter-by-millimeter in 8033 consecutive eyes. Arch. Ophthalmol..

[24] Shields C.L., Kaliki S., Furuta M., Fulco E., Alarcon C., Shields J.A. (2015). American Joint Committee on Cancer Classification of Uveal Melanoma (Anatomic Stage) Predicts Prognosis in 7,731 Patients: The 2013 Zimmerman Lecture. Ophthalmology.

[25] Djulbegovic M.B., Taylor D.J., Uversky V.N., Galor A., Shields C.L., Karp C.L. (2022). Intrinsic Disorder in BAP1 and Its Association with Uveal Melanoma. Genes.

[26] Harbour J.W., Onken M.D., Roberson E.D., Duan S., Cao L., Worley L.A., Council M.L., Matatall K.A., Helms C., Bowcock A.M. (2010). Frequent mutation of BAP1 in metastasizing uveal melanomas. Science.

[27] Glasgow B.J., McCannel T.A. (2018). Correlation of Immunocytochemistry of BRCA1-associated Protein-1 (BAP1) With Other Prognostic Markers in Uveal Melanoma. Am. J. Ophthalmol..

[28] Onken M.D., Worley L.A., Char D.H., Augsburger J.J., Correa Z.M., Nudleman E., Aaberg T.M., Altaweel M.M., Bardenstein D.S., Finger P.T. (2012). Collaborative Ocular Oncology Group report number 1: Prospective validation of a multi-gene prognostic assay in uveal melanoma. Ophthalmology.

[29] Onken M.D., Worley L.A., Ehlers J.P., Harbour J.W. (2004). Gene expression profiling in uveal melanoma reveals two molecular classes and predicts metastatic death. Cancer Res..

[30] Aaberg T.M., Covington K.R., Tsai T., Shildkrot Y., Plasseraud K.M., Alsina K.M., Oelschlager K.M., Monzon F.A. (2020). Gene Expression Profiling in Uveal Melanoma: Five-Year Prospective Outcomes and Meta-Analysis. Ocul. Oncol. Pathol..

[31] Plasseraud K.M., Cook R.W., Tsai T., Shildkrot Y., Middlebrook B., Maetzold D., Wilkinson J., Stone J., Johnson C., Oelschlager K. (2016). Clinical Performance and Management Outcomes with the DecisionDx-UM Gene Expression Profile Test in a Prospective Multicenter Study. J. Oncol..

