# Supplementary material for: Unexpectedly Low Rate of Metastasis and Death Among Patients Treated for Uveal Melanoma with Brachytherapy, Vitrectomy, and Silicone Oil
Source: Cancers (Basel). 2025 Aug 18;17(16):2683. doi: 10.3390/cancers17162683 (PMC12384185; doi:10.3390/cancers17162683)
Supplement: Supplementary file 1 [file cancers-17-02683-s001.zip › cancers-3756619-supplementary.pdf]

# Supplementary Materials: Unexpectedly Low Rate of Metastasis and Death Among Patients Treated for Uveal Melanoma with Brachytherapy, Vitrectomy, and Silicone Oil

**Axel Rivas , Wolfram Samlowski and Tara A. McCannel**

**Table S1.** Demographics.

| PN | Age | Gender | Race | Eye | CBI | ITH (mm) | IMD (mm) | T stage | M3 | DDx UM | CRS | PFS (Years) | OS (Years) | MET | Site of Met | Death |
|---|---|---|---|---|---|---|---|---|---|---|---|---|---|---|---|---|
| 1 | 73 | M | W | R | No | 3.58mm | 8.50mm | 1 | No | | Low | 8.70 | 8.70 | No | | No |
| 2 | 57 | M | H | R | No | 2.32mm | 7.50mm | 1 | No | | Low | 12.07 | 12.07 | No | | No |
| 4 | 71 | M | W | R | Yes | 12.01mm | 21.58mm | 4 | Yes | 2 | High | 1.57 | 1.82 | Yes | Liver | No |
| 5 | 70 | M | W | R | No | 3.78mm | Unknown | 1 | No | | Low | 6.18 | 6.18 | No | | No |
| 6 | 24 | F | W | L | No | 2.62mm | 10.2mm | 1 | No | 2 | High | 2.56 | 2.56 | No | | No |
| 8 | 61 | M | W | R | No | 3.20mm | Unknown | 1 | No | 1A | Low | 6.41 | 6.41 | No | | No |
| 10 | 62 | M | W | L | No | 6.05mm | 13.6mm | 2 | Yes | | High | 3.38 | 5.93 | Yes | Lung (L and R), liver | Yes |
| 11 | 54 | M | NA | L | Yes | 9.59mm | 13.22mm | 3 | Yes | | High | 9.88 | 9.88 | No | | No |
| 12 | 57 | F | W | L | No | 8.72mm | 13mm | 3 | No | 1A | Low | 5.13 | 5.13 | No | | No |
| 13 | 84 | F | W | R | No | 2.57mm | 12.99mm | 2 | | | | 0.27 | 0.27 | No | | No |
| 14 | 40 | M | H | R | No | 12.44mm | 18.98mm | 4 | No | 1A | Low | 1.60 | 1.60 | No | | No |
| 15 | 54 | M | W | L | No | 10.32mm | 21.62mm | 4 | | | | 0.69 | 0.69 | No | | No |
| 16 | 60 | M | W | L | No | 2.66mm | Unknown | 1 | | | | 0.41 | 0.41 | No | | No |
| 17 | 52 | M | W | L | No | 1.99mm | 6.88mm | 1 | No | 1A | Low | 5.97 | 5.97 | No | | No |
| 18 | 72 | F | W | L | No | 4.1mm | 11.3mm | 2 | Yes | | High | 2.44 | 3.99 | Yes | Liver, adrenal, bone (L pelvis) | No |
| 20 | 68 | M | W | R | No | 2.91mm | 8.3mm | 1 | Yes | | High | 10.60 | 10.60 | No | | No |
| 21 | 75 | F | W | L | No | 2.95mm | 8.07mm | 1 | No | 1A | Low | 3.71 | 3.71 | No | | No |
| 22 | 80 | M | W | L | No | 7.22mm | 18.81mm | 4 | No | | Low | 1.92 | 1.92 | No | | No |
| 23 | 58 | M | W | L | No | <3.0mm | 6.1-9.0mm | 1 | No | 1A | Low | 7.10 | 7.10 | No | | No |
| 24 | 52 | M | W | L | No | 6.10mm | 16.70mm | 3 | Yes | | High | 0.35 | 0.39 | Yes | Liver | No |
| 25 | 77 | M | W | R | No | 3.34mm | 9.99mm | 2 | No | | Low | 2.75 | 2.75 | No | | No |
| 26 | 51 | M | W | R | No | 1.14mm | 7.3mm | 1 | | | | 1.50 | 1.50 | No | | No |
| 29 | 66 | M | W | L | No | 14.92mm | 18.00mm | 4 | Yes | 2 | High | 0.18 | 0.18 | No | | No |
| 31 | 59 | F | W | R | No | 6.64mm | 10.33mm | 2 | Yes | 2 | High | 3.97 | 3.97 | No | | No |
| 32 | 56 | M | W | L | No | 3.49mm | Unknown | 1 | | | | 11.17 | 11.17 | No | | No |
| 33 | 70 | M | W | R | No | 8.48mm | 17.03mm | 3 | Yes | | High | 0.10 | 0.10 | No | | No |
| 35 | 57 | M | W | R | No | 3.15mm | 11.38mm | 2 | Yes | 2 | High | 5.22 | 5.22 | No | | No |
| 36 | 68 | M | W | L | No | 3.20mm | Unknown | 1 | | | | 1.50 | 1.50 | No | | No |
| 37 | 57 | M | W | L | No | 5.91mm | 14.77mm | 1 | Yes | | High | 13.66 | 13.66 | No | | No |
| 39 | 72 | M | W | L | No | 2.08mm | 8.88mm | 1 | No | | Low | 9.37 | 9.66 | Yes | Liver | No |
| 40 | 51 | F | W | L | Yes | 10.03mm | 14.27mm | 3 | Yes | 2 | High | 7.03 | 7.03 | No | | No |
| 42 | 57 | M | W | R | No | 2.23mm | 15.54mm | 2 | Yes | 1B | High | 1.69 | 1.69 | No | | No |
| 43 | 80 | M | W | L | Yes | 4.17mm | 12.43mm | 2 | Yes | 2 | High | 4.74 | 5.75 | Yes | Liver | No |
| 44 | 74 | F | W | R | Yes | 11.87mm | 15.96mm | 3 | | | | 0.04 | 0.04 | No | | No |
| 45 | 50 | F | NA | R | Yes | 5.81mm | 15.24mm | 3 | | 1A | Low | 3.38 | 3.38 | No | | No |
| 46 | 44 | F | W | R | No | 4.02mm | 12.13mm | 2 | Yes | 2 | High | 4.10 | 4.10 | No | | No |
| 47 | 48 | F | W | R | Yes | 11.14mm | 18.23mm | 3 | Yes | | High | 9.02 | 9.02 | No | | No |

PN: Patient number, F: Female, M: Male, W: White-Not Hispanic, H: White-Hispanic/Latino, A: Asian, NA: Not specified, L: Left Eye, R: Right Eye, CBI: Ciliary body involvement, ITH: Initial tumor height, IMD: Initial maximum diameter, M3: Monosomy 3, DDx UM: DecisionDx UM Score, CRS: Combined risk score, PFS: Progression free survival, OS: Overall survival, MET: Metastases, Site of Met: Site of metastases

**Table S2.** Demographic Summary.

| **Characteristics** | **N=47** |
|---|---|
| **Age (Years)** | |
| Median | 59 |
| Range | 24-84 |
| **Gender** | |
| Male | 26 (70%) |
| Female | 11 (30%) |
| **Race** | |
| White-Not Hispanic | 33 (90%) |
| White-Hispanic | 2 (5%) |
| Asian | 0 (0%) |
| Declined to Specify | 2 (5%) |

**Table S3.** T Stage Summary.

| **Characteristics** | **T1 Stage** | **T2 Stage** | **T3 Stage** | **T4 Stage** |
|---|---|---|---|---|
| **Number of Individuals (*n*=)** | 15 | 9 | 8 | 5 |
| **Age (Years)** | | | | |
| Mean Patient Age | 60.13±12.66 | 65.78±13.17 | 57±9.70 | 62.2±15.56 |
| Median | 60 | 62 | 53 | 53 |
| Range | 24-75 | 44-84 | 48-74 | 40-80 |
| **Gender** | | | | |
| Male | 13 | 5 | 3 | 5 |
| Female | 2 | 4 | 5 | 0 |
| **Tumor size (mm)** | | | | |
| Average height | 3.01±1.07 | 4.03±1.48 | 8.97±2.18 | 11.38±2.85 |
| Average maximum diameter | 8.84±2.17 | 12.19±1.72 | 15.46±1.87 | 19.79±1.69 |
| **Progression Free Survival (Years)** | | | | |
| Average PFS | 6.41±4.18 | 3.17±1.56 | 4.37±4.03 | 1.19±0.73 |
| Range | 0.41-13.66 | 0.27-5.22 | 0.04-9.88 | 0.18-1.92 |
| **Overall Survival (Years)** | | | | |
| Average OS | 6.41±4.19 | 3.74±1.89 | 4.37±4.03 | 1.24±0.77 |
| Range | 0.41-13.66 | 0.27-5.93 | 0.04-9.88 | 0.18-1.92 |
| **Individuals Progressing to Metastasis** | 1 | 3 | 1 | 1 |
| **Individuals Progressing to Death** | 0 | 1 | 0 | 0 |

**Table S4.** Chromosome 3 Summary.

| **Characteristics** | **Monosomy 3** | **Disomy 3** |
|---|---|---|
| **Number of Individuals (*n*=)** | 16 | 13 |
| **Age (Years)** | | |
| Mean Patient Age | 60.5±9.91 | 61.23±16.05 |
| Median | 58 | 61 |
| Range | 44-80 | 24-80 |
| **Gender** | | |
| Male | 11 | 10 |
| Female | 5 | 3 |
| **Tumor size (mm)** | | |
| Average height | 6.97±3.70 | 4.52±3.24 |
| Average maximum diameter | 14.30±3.43 | 10.89±4.28 |

| | | |
|---|---|---|
| **Progression Free Survival (Years)** | | |
| Average PFS | 4.87±4.11 | 5.65±3.16 |
| Range | 0.10-13.66 | 1.6-12.07 |
| **Overall Survival (Years)** | | |
| Average OS | 5.21±4.03 | 5.67±3.19 |
| Range | 0.101-13.66 | 1.6-12.07 |
| **Individuals Progressing to Metastasis** | 5 | 1 |
| **Individuals Progressing to Death** | 1 | 0 |

**Table S5.** Decision Dx UM Summary.

| Characteristics | DecisionDx UM Class 1A | DecisionDx UM Class 1B | DecisionDx UM Class 2 |
|---|---|---|---|
| Number of Individuals (*n*=) | 7 | 1 | 8 |
| **Age (Years)** | | | |
| Mean Patient Age | 56.14±10.79 | 57 | 56.5±17.33 |
| Median | 57 | | 58 |
| Range | 40-75 | | 24-80 |
| **Gender** | | | |
| Male | 4 | 1 | 4 |
| Female | 3 | 0 | 4 |
| Monosomy 3 Individuals | 0 | 1 | 7 |
| **Tumor size (mm)** | | | |
| Average height | 5.85±4.05 | 2.23 | 7.19±4.59 |
| Average maximum diameter | 11.86±4.71 | 15.54 | 13.79±4.03 |
| **Progression Free Survival (Years)** | | | |
| Average PFS | 4.76±1.95 | 1.69 | 3.67±2.17 |
| Range | 1.6-7.10 | | 0.18-7.03 |
| **Overall Survival (Years)** | | | |
| Average OS | 4.76±1.95 | 1.69 | 3.83±2.23 |
| Range | 1.6-7.10 | | 0.18-7.03 |
| Individuals Progressing to Metastasis | 0 | 0 | 2 |
| Individuals Progressing to Death | 0 | 0 | 0 |
